# Extracellular and ER-stored Ca^2+^ contribute to BIRD-2-induced cell death in diffuse large B-cell lymphoma cells

**DOI:** 10.1038/s41420-018-0118-6

**Published:** 2018-11-02

**Authors:** Mart Bittremieux, Rita M. La Rovere, Marleen Schuermans, Tomas Luyten, Katsuhiko Mikoshiba, Peter Vangheluwe, Jan B. Parys, Geert Bultynck

**Affiliations:** 1 Laboratory of Molecular and Cellular Signaling, Department of Cellular and Molecular Medicine, KU Leuven and Leuven Kanker Instituut, Leuven, 3000 Belgium; 2 Laboratory of Cellular Transport Systems, Department of Cellular and Molecular Medicine, KU Leuven, Leuven, 3000 Belgium; 3 The Laboratory for Developmental Neurobiology, Brain Science Institute, RIKEN, 2-1 Hirosawa, Wako, Saitama, 351-0198 Japan

## Abstract

The anti-apoptotic protein Bcl-2 is upregulated in several cancers, including diffuse large B-cell lymphoma (DLBCL) and chronic lymphocytic leukemia (CLL). In a subset of these cancer cells, Bcl-2 blocks Ca^2+^-mediated apoptosis by suppressing the function of inositol 1,4,5-trisphosphate (IP_3_) receptors (IP_3_Rs) located at the endoplasmic reticulum (ER). A peptide tool, called Bcl-2/IP_3_ receptor disruptor-2 (BIRD-2), was developed to disrupt Bcl-2/IP_3_R complexes, triggering pro-apoptotic Ca^2+^ signals and killing Bcl-2-dependent cancer cells. In DLBCL cells, BIRD-2 sensitivity depended on the expression level of IP_3_R2 channels and constitutive IP_3_ signaling downstream of the B-cell receptor. However, other cellular pathways probably also contribute to BIRD-2-provoked cell death. Here, we examined whether BIRD-2-induced apoptosis depended on extracellular Ca^2+^ and more particularly on store-operated Ca^2+^ entry (SOCE), a Ca^2+^-influx pathway activated upon ER-store depletion. Excitingly, DPB162-AE, a SOCE inhibitor, suppressed BIRD-2-induced cell death in DLBCL cells. However, DPB162-AE not only inhibits SOCE but also depletes the ER Ca^2+^ store. Treatment of the cells with YM-58483 and GSK-7975A, two selective SOCE inhibitors, did not protect against BIRD-2-induced apoptosis. Similar data were obtained by knocking down STIM1 using small interfering RNA. Yet, extracellular Ca^2+^ contributed to BIRD-2 sensitivity in DLBCL, since the extracellular Ca^2+^ buffer ethylene glycol tetraacetic acid (EGTA) blunted BIRD-2-triggered apoptosis. The protective effects observed with DPB162-AE are likely due to ER Ca^2+^-store depletion, since a similar protective effect could be obtained using the sarco/endoplasmic reticulum Ca^2+^-ATPase inhibitor thapsigargin. Thus, both the ER Ca^2+^-store content and extracellular Ca^2+^, but not SOCE, are critical factors underlying BIRD-2-provoked cell death.

## Introduction

Cell death and survival is regulated by the Bcl-2-protein family, which consists of pro-apoptotic and anti-apoptotic family members^1^. The anti-apoptotic protein Bcl-2 is upregulated in a large number of cancer cells, including B-cell lymphomas like chronic lymphocytic leukemia (CLL) and diffuse large B-cell lymphoma (DLBCL)^2,3^. Bcl-2 prevents apoptotic cell death by neutralizing pro-apoptotic family members, including the executioner proteins Bak and Bax and the BH3-only protein Bim, at the mitochondria^4,5^. BH3-mimetic compounds, like venetoclax, disrupt the binding between Bcl-2 and pro-apoptotic BH3-only proteins, thereby triggering apoptotic cell death in cancer cells that depend on Bcl-2's function at the mitochondria for their survival^6,7^. Furthermore, the Bcl-2 protein is also located at the endoplasmic reticulum (ER), the main intracellular Ca^2+^ store^8,9^. There, Bcl-2 binds with its Bcl-2 homology 4 (BH4) domain to the central, modulatory domain of the inositol 1,4,5-trisphosphate (IP_3_) receptor (IP_3_R)^10^. In this way, Bcl-2 blocks excessive, pro-apoptotic, IP_3_R-mediated Ca^2+^ release from the ER, thereby preventing mitochondrial Ca^2+^ overload and subsequent apoptotic cell death^10^. Based on the binding site of Bcl-2 on the IP_3_R, a peptide tool was developed in an attempt to target pro-survival Bcl-2 proteins at the ER in cancer cells^11^. This cell-permeable peptide, called Bcl-2/IP_3_R disruptor-2 (BIRD-2), is capable of stripping Bcl-2 from the IP_3_R, without affecting Bcl-2/Bim complexes. BIRD-2 was shown to kill Bcl-2-dependent cancer cells, like DLBCL and CLL cells, by eliciting spontaneous, pro-apoptotic Ca^2+^ signals^12,13^. On the other hand, the survival of normal peripheral mononuclear blood cells was not affected by the peptide tool. Furthermore, follicular lymphoma and small-cell lung cancer cells could be killed by BIRD-2 as well and the peptide even decreased the in vivo tumor growth of human myeloma cells in xenografted mouse models^14,15^. Interestingly, in DLBCL cells BIRD-2 sensitivity correlated to the expression level of isoform 2 of the IP_3_R, which is the isoform with the highest sensitivity towards its ligand IP_3_^12^. DLBCL cells with high IP_3_R2 levels, like SU-DHL-4 cells, were very sensitive to BIRD-2, whereas cells with low IP_3_R2 expression levels, such as OCI-LY-1, appeared to be rather resistant to the peptide. On the other hand, OCI-LY-1 cells are very sensitive to BH3-mimetic drugs, like venetoclax^16^. Recent work from our group showed that there exists an opposite correlation between the susceptibility of DLBCL cells to BIRD-2 and venetoclax^16^. Additionally, constitutive IP_3_ signaling also underlies BIRD-2 sensitivity in B-cell cancers^17^. DLBCL and primary CLL cells could be protected from BIRD-2-triggered apoptosis by blocking constitutive phospholipase C and IP_3_ signaling.

However, it is not clear whether other cellular factors contribute to BIRD-2-induced cell death in cancer cells. In particular, we found that BIRD-2 provoked spontaneous Ca^2+^ oscillations in B-cell malignancies^13^, which eventually result in Ca^2+^ overload via IP_3_R-mediated Ca^2+^ fluxes^12^. In many cells, Ca^2+^ oscillations are maintained through the concerted action of Ca^2+^ release from the ER and Ca^2+^ influx from the extracellular milieu. Therefore, we assessed whether extracellular Ca^2+^ and Ca^2+^ entry mechanisms such as store-operated Ca^2+^ entry (SOCE) contributed to BIRD-2 cytotoxicity. SOCE is an important Ca^2+^-influx pathway that is activated upon ER-store depletion^18^. It is mediated through STIM and Orai proteins^19–23^. STIM proteins are present in the ER membrane where they serve as luminal Ca^2+^ sensors, while Orai proteins are located in the plasma membrane and function as Ca^2+^-influx channels^20–22^. Upon depletion of the ER Ca^2+^ store, STIM1 proteins form oligomers that translocate to parts of the ER membrane that are in close contact with the plasma membrane. There, STIM1 activates Orai1 channels to trigger Ca^2+^ influx^23,24^. The BIRD-2 peptide provokes IP_3_-induced Ca^2+^ release in cancer cells that depend for their survival on the function of Bcl-2 at the ER. Hence, we hypothesized that BIRD-2 treatment results in SOCE activation, subsequently enabling Ca^2+^ influx that is contributing to intracellular Ca^2+^ overload and thus cell death. Here, we focused on SU-DHL-4 cells to test our hypothesis, since this DLBCL cell model displays the highest sensitivity towards BIRD-2^12,16^. Our data indicate that despite the fact that extracellular Ca^2+^ is important for BIRD-2-induced cell death in these cells, SOCE was dispensable as pharmacological SOCE inhibitors YM-58483 and GSK-7975A and Stim1 knockdown failed to protect against BIRD-2-induced apoptosis. Interestingly, DPB162-AE, a SOCE inhibitor that also depletes the ER Ca^2+^ stores, did protect against BIRD-2-induced cell death. However, this is likely through its action at the ER, since ER Ca^2+^-store depletion brought about by the sarco/ER Ca^2+^-ATPase (SERCA) inhibitor thapsigargin (TG) too suppressed BIRD-2 toxicity.

## Results

### BIRD-2-induced cell death in SU-DHL-4 cells is reduced by DPB162-AE, a SOCE inhibitor that also depletes the ER Ca^2+^ store

**Fig. 1:**
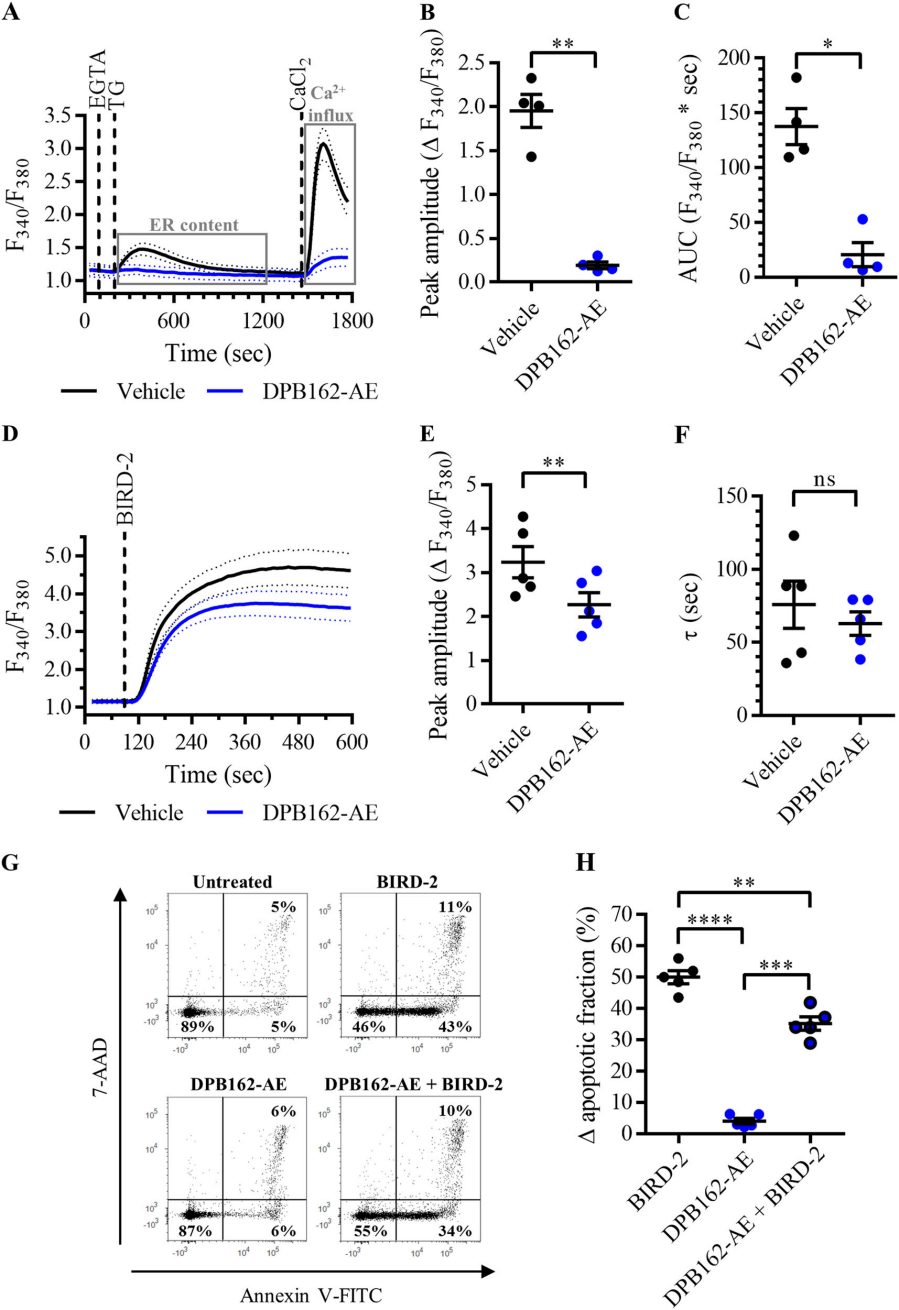
DPB162-AE reduces the cytotoxic effects of BIRD-2 in SU-DHL-4 cells. **a** Analysis of Ca^2+^ influx after store depletion in SU-DHL-4 cells using the ratiometric Ca^2+^ indicator Fura-2 AM. Cells were pre-treated for 30 min with vehicle (black line) or with 10 µM DPB162-AE (blue line). To deplete the ER Ca^2+^ store, 3 mM EGTA and 1 µM thapsigargin (TG) were added as indicated. After store depletion, Ca^2+^ influx was triggered by adding 10 mM CaCl_2_. The curves represent the mean ± SEM of four independent experiments. Quantification of the Ca^2+^ influx is provided as the peak amplitude (∆ *F*_340_/*F*_380_) in **b**, whereas the TG-releasable Ca^2+^ is quantified as the AUC (*F*_340_/*F*_380_ × s) in **c**. **d** Analysis of the cytosolic Ca^2+^ response in SU-DHL-4 cells using Fura-2 AM. Cells were pre-treated for 30 min with vehicle (black line) or 10 µM DPB162-AE (blue line). Addition of 10 µM BIRD-2 is indicated by the dotted line. The curves represent the mean ± SEM of five independent experiments. The BIRD-2-provoked cytosolic Ca^2+^ rise is quantified by measuring the peak amplitude (∆ *F*_340_/*F*_380_), shown in **e**, and the time constant *τ* (s), which is shown in **f**. **g** Representative scatter plots from flow cytometry analysis detecting apoptosis in SU-DHL-4 cells stained with Annexin V-FITC and 7-AAD. Cells were pre-treated with or without 10 µM DPB162-AE 30 min prior to application of 10 µM BIRD-2. After 2 h of BIRD-2 treatment, apoptotic cell death was detected by measuring the Annexin V-FITC-positive fraction. **h** Quantitative analysis of five independent experiments detecting apoptosis in SU-DHL-4 cells treated for 2 h with 10 µM BIRD-2, with or without pre-treatment with 10 µM DPB162-AE. The ∆ apoptotic fraction is plotted, which corresponds to the apoptotic fraction corrected for the percentage of apoptosis detected in untreated cells. In the dot plots, data are represented as the mean ± SEM of at least four independent experiments. Statistically significant differences were determined with a paired two-tailed Student’s *t* test or a one-way ANOVA, as appropriate (*p < 0.05, **p < 0.01, ***p < 0.001, ****p < 0.0001). NS not significant

In DLBCL cell lines, BIRD-2 potently induces apoptotic cell death by disrupting Bcl-2/IP_3_R complexes, resulting in excessive Ca^2+^ signaling^12,16^. Since BIRD-2 triggers Ca^2+^ release from the ER, we wondered whether SOCE occurs upon BIRD-2 application and thus contributes to the cell death-inducing properties of the peptide tool. We explored this hypothesis by focusing on the BIRD-2-sensitive DLBCL cell model, SU-DHL-4^16^, in which we studied the impact of the SOCE inhibitor DPB162-AE on the BIRD-2-induced Ca^2+^ rise and cell death response. In this cell model, DPB162-AE acts as a potent SOCE inhibitor (Fig. 1a), confirming previously published data^25^. Pre-treatment of Fura-2 AM-loaded SU-DHL-4 cells with 10 µM DPB162-AE for 30 min reduced Ca^2+^ influx after store depletion (Fig. 1a). Ca^2+^ influx was quantified by measuring the peak amplitude. Compared to the control condition, the peak amplitude was significantly reduced in cells pre-treated with DPB162-AE (Fig. 1b). However, in accordance with our previous findings^25^, DPB162-AE did not only inhibit SOCE, but also depleted the ER Ca^2+^ store (Fig. 1a). The TG-releasable Ca^2+^ was quantified by measuring the area under the curve (AUC), which was significantly reduced in cells pre-treated with DPB162-AE (Fig. 1c). Next, it was determined whether DPB162-AE treatment has an effect on the pro-apoptotic, cytosolic Ca^2+^ rise triggered by BIRD-2 addition. Therefore, SU-DHL-4 cells were loaded with Fura-2 AM and pre-treated for 30 min with 10 µM DPB162-AE. Subsequently, BIRD-2, at a concentration of 10 µM which is the IC_50_ value of the peptide in SU-DHL-4 cells^16^, was added while monitoring the cytosolic Ca^2+^ levels (Fig. 1d). Compared to the control condition, the BIRD-2-provoked Ca^2+^ rise was reduced in cells pre-treated with DPB162-AE. The cytosolic Ca^2+^ response was quantified by measuring the peak amplitude and the time constant *τ*. Compared to cells pre-treated with vehicle, the peak amplitude was significantly reduced in DPB162-AE-pre-treated cells (Fig. 1e). The time constant *τ* also displayed a tendency to be lower in DPB162-AE-pre-treated cells, although there was no significant difference with the control condition (Fig. 1f). Then, it was investigated whether DPB162-AE also reduces BIRD-2-triggered apoptotic cell death. To do so, SU-DHL-4 cells were pre-treated for 30 min with 10 µM DPB162-AE, followed by treatment with 10 µM BIRD-2. After 2h of peptide treatment, cell death was measured via Annexin V-FITC and 7-aminoactinomycin D (7-AAD) staining of the cells (Fig. 1g). BIRD-2 induced approximately 50% of apoptotic cell death in the SU-DHL-4 cells, whereas treatment with DPB162-AE was only slightly toxic (~5% apoptosis) for the cells (Fig. 1h). Interestingly, DPB162-AE pre-treatment significantly reduced BIRD-2-triggered apoptosis compared to cells treated with BIRD-2 alone. These results indicate that DPB162-AE protects against BIRD-2-provoked cell death, although we do not know whether this is due to its inhibitory effect on SOCE or due to depletion of the ER Ca^2+^ store.

### Selective inhibition of SOCE with pharmacological compounds does not protect against BIRD-2-triggered cell death

**Fig. 2:**
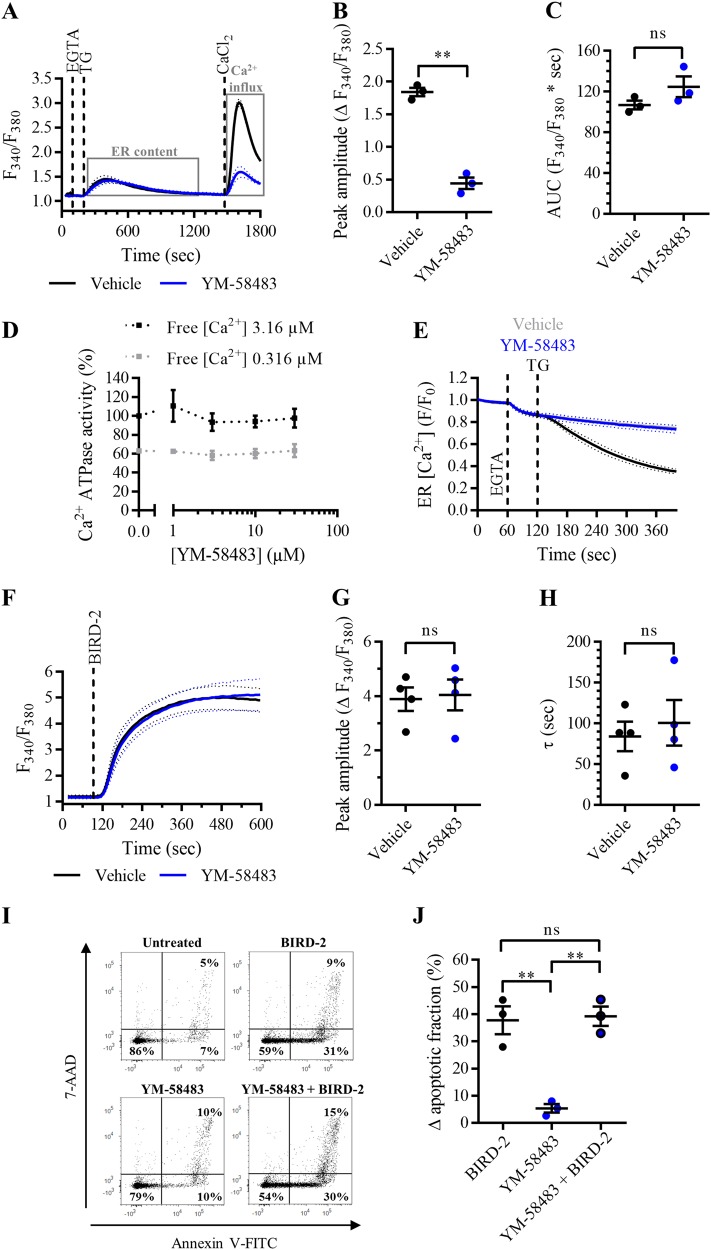
SOCE inhibition with YM-58483 does not reduce cell death triggered by BIRD-2 in SU-DHL-4 cells. **a** Analysis of Ca^2+^ influx after store depletion in SU-DHL-4 cells using the ratiometric Ca^2+^ indicator Fura-2 AM. Cells were pre-treated for 30 min with vehicle (black line) or with 3 µM YM-58483 (blue line). To deplete the ER Ca^2+^ store, 3 mM EGTA and 1 µM TG were added as indicated. After store depletion, Ca^2+^ influx was triggered by adding 10 mM CaCl_2_. The curves represent the mean ± SEM of three independent experiments. Quantification of the Ca^2+^ influx is provided as the peak amplitude (∆ *F*_340_/*F*_380_) in **b**, whereas the TG-releasable Ca^2+^ is quantified as the AUC (*F*_340_/*F*_380_ × s) in **c**. **d** Dose–response curve of YM-58483 on SERCA2b ATPase activity (%). The Ca^2+^-dependent ATPase activity was measured at maximal (free [Ca^2+^] 3.16 µM) and submaximal (free [Ca^2+^] 0.316 µM) [Ca^2+^] for different treatments, including vehicle (control) and different [YM-58483]. Data were normalized to the values obtained in the control condition at maximal [Ca^2+^], which was set at 100%. Data are represented at the mean ± SEM of three independent experiments. **e** Single-cell analysis of the ER Ca^2+^ levels in HeLa cells transfected with G-CEPIA1*er* plasmid. Cells were treated with vehicle (gray curve), 1 µM TG (black curve), or 3 µM YM-58483 (blue curve) 60 s after the addition of 3 mM EGTA. Data are represented as the mean ± SEM of three independent experiments (*n* > 100 cells/condition). **f** Analysis of the cytosolic Ca^2+^ response in SU-DHL-4 cells using Fura-2 AM. Cells were pre-treated for 30 min with vehicle (black line) or 3 µM YM-58483 (blue line). Addition of 10 µM BIRD-2 is indicated by the dotted line. The curves represent the mean ± SEM of four independent experiments. The BIRD-2-provoked cytosolic Ca^2+^ rise is quantified by measuring the peak amplitude (∆ *F*_340_/*F*_380_), shown in **g**, and the time constant *τ* (s), which is shown in **h**. **i** Representative scatter plots from flow cytometry analysis detecting apoptosis in SU-DHL-4 cells stained with Annexin V-FITC and 7-AAD. Cells were pre-treated with or without 3 µM YM-58483 30 min prior to application of 10 µM BIRD-2. After 2 h of BIRD-2 treatment, apoptotic cell death was detected by measuring the Annexin V-FITC-positive fraction. **j** Quantitative analysis of three independent experiments detecting apoptosis in SU-DHL-4 cells treated for 2 h with 10 µM BIRD-2, with or without pre-treatment with 3 µM YM-58483. The ∆ apoptotic fraction is plotted, which corresponds to the apoptotic fraction corrected for the percentage of apoptosis detected in untreated cells. In the dot plots, data are represented as the mean ± SEM of at least three independent experiments. Statistically significant differences were determined with a paired two-tailed Student’s *t* test or a one-way ANOVA, as appropriate (**p < 0.01). NS not significant.

**Fig. 3:**
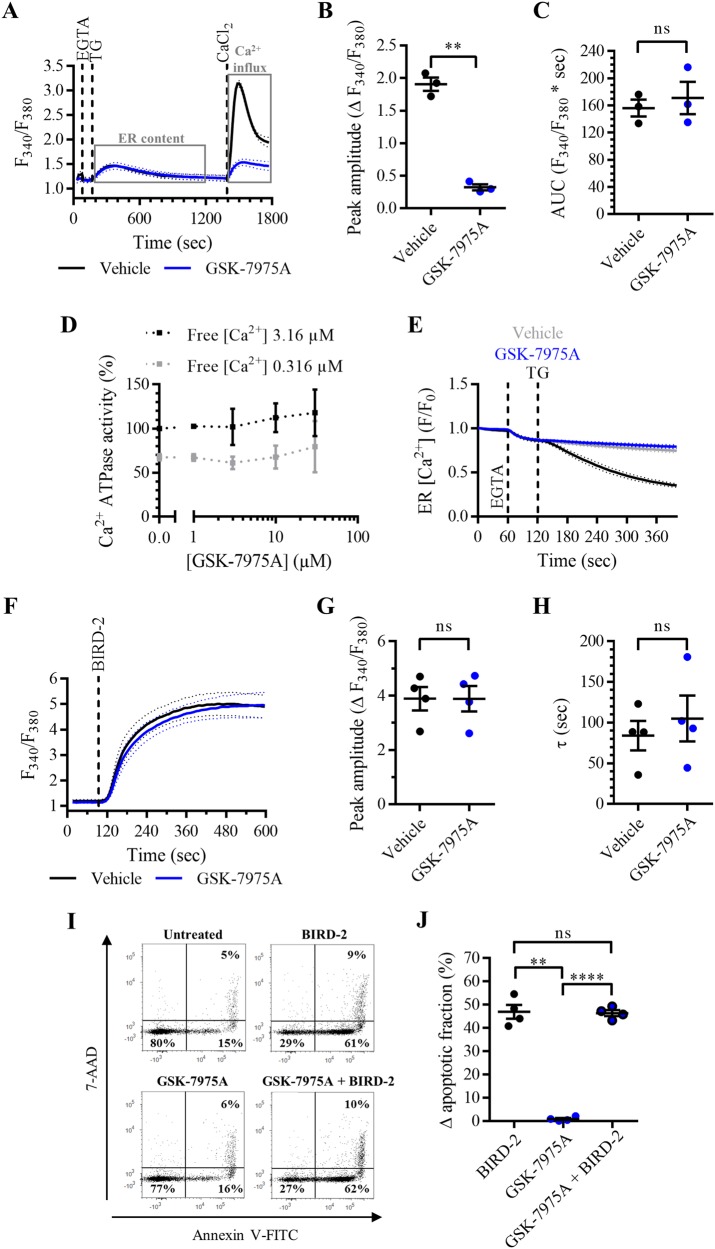
SOCE inhibition with GSK-7975A does not reduce apoptosis induced by BIRD-2 in SU-DHL-4 cells. **a** Analysis of Ca^2+^ influx after store depletion in SU-DHL-4 cells using the ratiometric Ca^2+^ indicator Fura-2 AM. Cells were pre-treated for 30 min with vehicle (black line) or with 3 µM GSK-7975A (blue line). To deplete the ER Ca^2+^ store, 3 mM EGTA and 1 µM TG were added as indicated. After store depletion, Ca^2+^ influx was triggered by adding 10 mM CaCl_2_. The curves represent the mean ± SEM of three independent experiments. Quantification of the Ca^2+^ influx is provided as the peak amplitude (∆ *F*_340_/*F*_380_) in **b**, whereas the TG-releasable Ca^2+^ is quantified as the AUC (*F*_340_/*F*_380_ × s) in **c**. **d** Dose–response curve of GSK-7975A on SERCA2b ATPase activity (%). The Ca^2+^-dependent ATPase activity was measured at maximal (free [Ca^2+^] 3.16 µM) and submaximal (free [Ca^2+^] 0.316 µM) [Ca^2+^] for different treatments, including vehicle (control) and different [GSK-7975A]. Data were normalized to the values obtained in the control condition at maximal [Ca^2+^], which was set at 100%. Data are represented at the mean ± SEM of three independent experiments. **e** Single-cell analysis of the ER Ca^2+^ levels in HeLa cells transfected with G-CEPIA1*er* plasmid. Cells were treated with vehicle (gray curve), 1 µM TG (black curve), or 3 µM GSK-7975A (blue curve) 60 s after the addition of 3 mM EGTA. Data are represented as the mean ± SEM of three independent experiments (*n* > 100 cells/condition). **f** Analysis of the cytosolic Ca^2+^ response in SU-DHL-4 cells using Fura-2 AM. Cells were pre-treated for 30 min with vehicle (black line) or 3 µM GSK-7975A (blue line). Addition of 10 µM BIRD-2 is indicated by the dotted line. The curves represent the mean ± SEM of four independent experiments. The BIRD-2-provoked cytosolic Ca^2+^ rise is quantified by measuring the peak amplitude (∆ *F*_340_/*F*_380_), shown in **g**, and the time constant *τ* (s), which is shown in **h**. **i** Representative scatter plots from flow cytometry analysis detecting apoptosis in SU-DHL-4 cells stained with Annexin V-FITC and 7-AAD. Cells were pre-treated with or without 3 µM GSK-7975A 30 min prior to application of 10 µM BIRD-2. After 2 h of BIRD-2 treatment, apoptotic cell death was detected by measuring the Annexin V-FITC-positive fraction. **j** Quantitative analysis of four independent experiments detecting apoptosis in SU-DHL-4 cells treated for 2 h with 10 µM BIRD-2, with or without pre-treatment with 3 µM GSK-7975A. The ∆ apoptotic fraction is plotted, which corresponds to the apoptotic fraction corrected for the percentage of apoptosis detected in untreated cells. In the dot plots, data are represented as the mean ± SEM of at least three independent experiments. Statistically significant differences were determined with a paired two-tailed Student’s *t* test or a one-way ANOVA, as appropriate (**p < 0.01, ****p < 0.0001). NS not significant.

Pharmacological tools that selectively inhibit SOCE were used to test whether DPB162-AE treatment confers protection to BIRD-2 via inhibition of the Ca^2+^-influx pathway. For this purpose, YM-58483 and GSK-7975A were used. First, it was validated that YM-58483 selectively inhibited SOCE. Compared to vehicle-pre-treated cells, Ca^2+^ influx after store depletion was reduced in SU-DHL-4 cells pre-treated with 3 µM YM-58483 (Fig. 2a). SOCE was quantified by measuring the peak amplitude, which was significantly reduced in YM-58483-pre-treated cells (Fig. 2b). Moreover, YM-58483 did not alter the ER Ca^2+^ content, since there was no significant difference in the AUC between YM-58483-pre-treated and vehicle-pre-treated cells (Fig. 2c). To ascertain that YM-58483 has no effect on the ER Ca^2+^ levels, it was determined whether the compound affects ER Ca^2+^ uptake by measuring the ATPase activity of SERCA2b, the house-keeping SERCA isoform (Fig. 2d), and whether it affects ER Ca^2+^ leak by directly measuring ER Ca^2+^ levels upon YM-58483 application (Fig. 2e). First, SERCA2b ATPase activity was measured in the presence of increasing concentrations of YM-58483 at submaximal (free [Ca^2+^] 0.316 µM) and maximal (free [Ca^2+^] 3.16 µM) [Ca^2+^] in COS microsomes overexpressing SERCA2b (Fig. 2d). Compared to the control condition, YM-58483 does not alter SERCA2b ATPase activity at maximal and submaximal [Ca^2+^]. Furthermore, it was examined whether the SOCE inhibitor induces an ER Ca^2+^-leak pathway by measuring the ER Ca^2+^ levels in HeLa cells transfected with the G-CEPIA1*er* plasmid^26^. ER Ca^2+^ levels were decreased upon application of 1 µM TG, which inhibits the SERCA pump (Fig. 2e). On the other hand, acute addition of 3 µM YM-58483 did not reduce ER Ca^2+^ levels. These results indicate that YM-58483 has no effect on the ER Ca^2+^-store content and it can therefore be used as a selective inhibitor of SOCE. Next, it was determined whether inhibiting SOCE with YM-58483 protects against BIRD-2 cytotoxicity in SU-DHL-4 cells. The cytosolic Ca^2+^ rise provoked by the addition of 10 µM BIRD-2 was measured in SU-DHL-4 cells pre-treated with vehicle or 3 µM YM-58483. Strikingly, the BIRD-2-triggered Ca^2+^ rise was comparable between cells treated with YM-58483 and the control condition (Fig. 2f). The cytosolic Ca^2+^ response was quantified by measuring the peak amplitude and the time constant *τ*. There was neither a significant difference between the peak amplitude (Fig. 2g) nor the time constant *τ* (Fig. 2h) of YM-58483-pre-treated and vehicle-pre-treated SU-DHL-4 cells. Furthermore, apoptotic cell death triggered by 10 µM BIRD-2 was not reduced in SU-DHL-4 cells that were pre-treated for 30 min with 3 µM YM-58483, compared to cells treated with BIRD-2 alone (Fig. 2i). BIRD-2 treatment as well as YM-58483 + BIRD-2 treatment induced approximately 40% of apoptotic cell death, while treatment of the cells with the SOCE inhibitor alone was only slightly toxic (~5% apoptosis) for the cells (Fig. 2j). These results already strongly suggest that SOCE is not activated by BIRD-2 in the SU-DHL-4 DLBCL cells. Furthermore, these results were confirmed by a second pharmacological SOCE inhibitor, GSK-7975A. Treatment of SU-DHL-4 cells with 3 µM GSK-7975A potently inhibited Ca^2+^ influx after store depletion (Fig. 3a, b). GSK-7975A is a selective inhibitor of SOCE, as this compound neither reduced the ER Ca^2+^ content (Fig. 3a–c) nor affected SERCA2b ATPase activity (Fig. 3d) nor altered the ER Ca^2+^ levels (Fig. 3e). Similarly to YM-58483, GSK-7975A did not reduce the cytosolic Ca^2+^ rise induced by 10 µM BIRD-2 addition to SU-DHL-4 cells (Fig. 3f). The peak amplitude (Fig. 3g) and the time constant *τ* (Fig. 3h) were comparable between vehicle-treated and GSK-7975A-treated cells. Finally, apoptotic cell death triggered by 10 µM BIRD-2 was not reduced by inhibiting SOCE with GSK-7975A (Fig. 3i). Of note, GSK-7975A by itself was absolutely not toxic for the SU-DHL-4 cells. Hence, BIRD-2 cytotoxicity was comparable between cells treated with peptide alone or with GSK-7975A in combination with BIRD-2 (Fig. 3j). In summary, these results indicate that BIRD-2 does not depend on Ca^2+^ influx through SOCE for its pro-apoptotic effects in SU-DHL-4 DLBCL cells.

### Knockdown of STIM1 with siRNA does not confer protection against BIRD-2-induced apoptosis in SU-DHL-4 cells

**Fig. 4:**
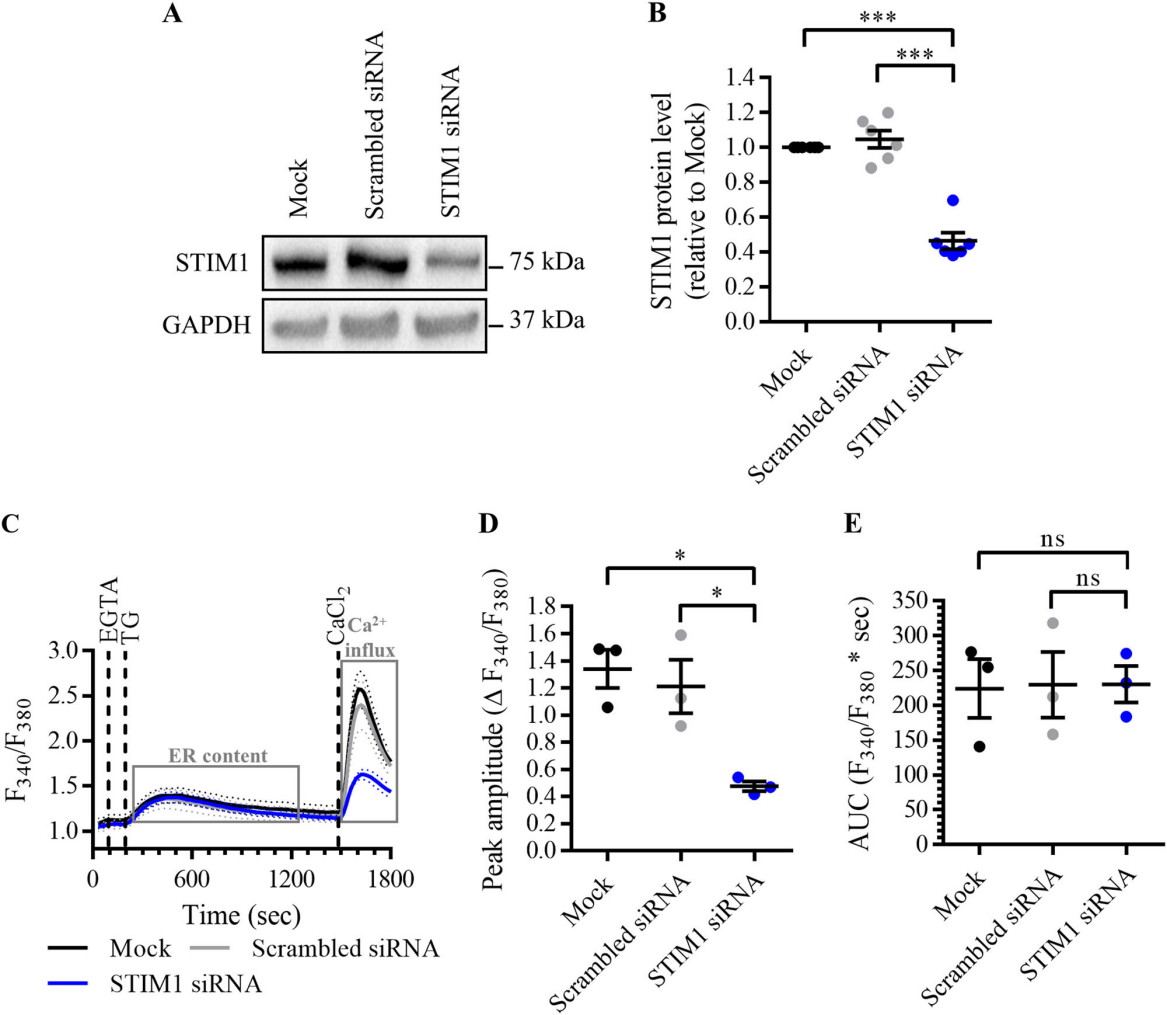
Knockdown of STIM1 with siRNA inhibits SOCE without depleting the ER Ca^2+^ store. **a** A representative western blot of six independent experiments showing the expression level of STIM1 in mock-transfected SU-DHL-4 cells, or cells transfected with scrambled siRNA or with siRNA against STIM1. The expression level of GAPDH was used as loading control. **b** Quantification of the STIM1/GAPDH protein levels of six independent experiments relative to the level in mock-transfected SU-DHL-4 cells, which was set at 1. **c** Analysis of Ca^2+^ influx after store depletion in transfected SU-DHL-4 cells using the ratiometric Ca^2+^ indicator Fura-2 AM. To deplete the ER Ca^2+^ store, 3 mM EGTA and 1 µM TG were added as indicated. After store depletion, Ca^2+^ influx was triggered by adding 10 mM CaCl_2_. The curves represent the mean ± SEM of three independent experiments. Quantification of the Ca^2+^ influx is provided as the peak amplitude (∆ *F*_340_/*F*_380_) in **d**, whereas the TG-releasable Ca^2+^ is quantified as the AUC (*F*_340_/*F*_380 _× s) in **e**. In the dot plots, data are represented as the mean ± SEM of at least three independent experiments. Statistically significant differences were determined with a one-way ANOVA (*p < 0.05, ***p < 0.001). NS not significant.

**Fig. 5:**
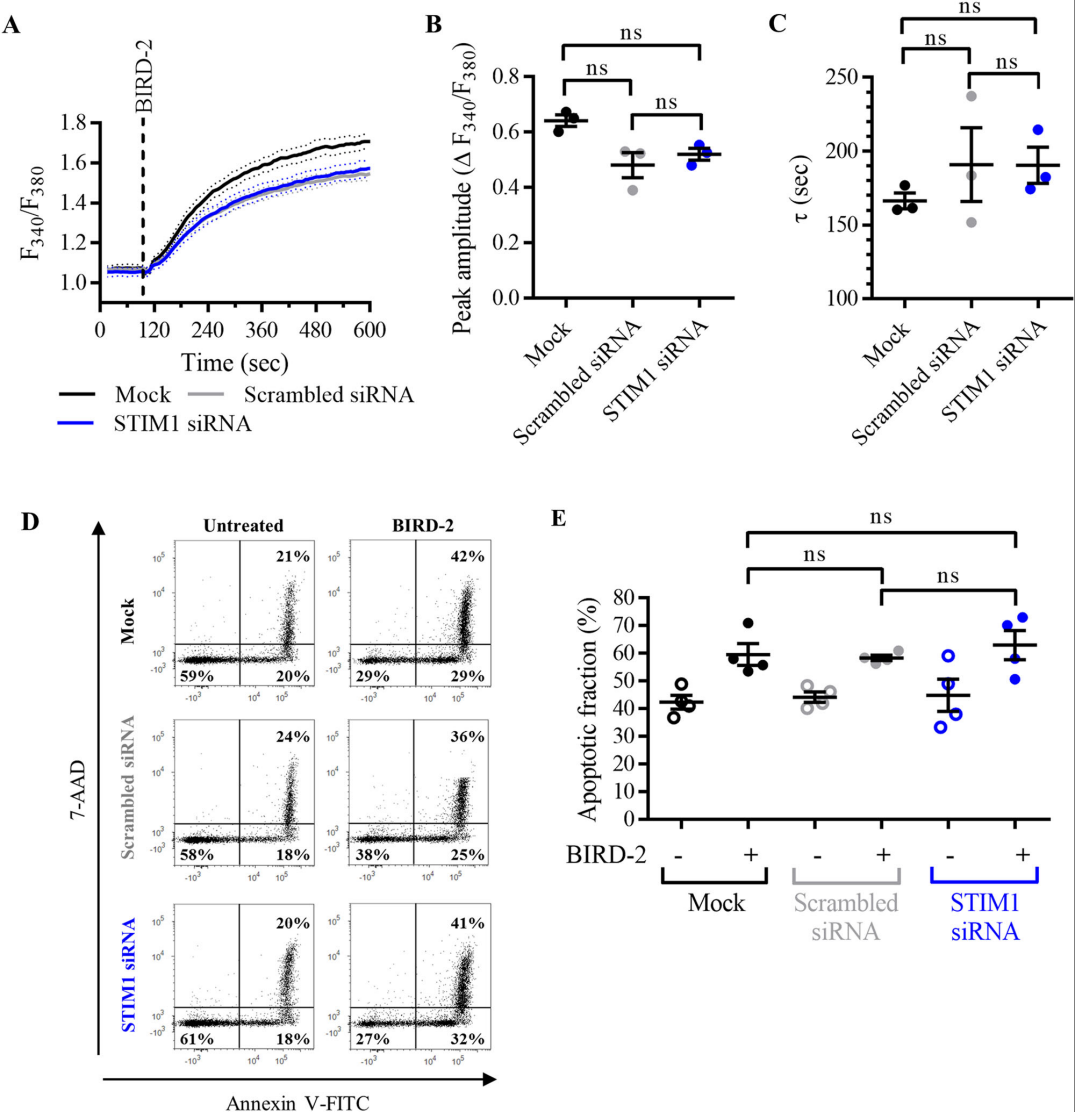
Knockdown of STIM1 with siRNA does not reduce apoptosis triggered by BIRD-2 in SU-DHL-4 cells. **a** Analysis of the cytosolic Ca^2+^ response in mock-transfected SU-DHL-4 cells, or cells transfected with scrambled siRNA or with siRNA against STIM1, using Fura-2 AM. Addition of 10 µM BIRD-2 is indicated by the dotted line. The curves represent the mean ± SEM of three independent experiments. The BIRD-2-provoked cytosolic Ca^2+^ rise is quantified by measuring the peak amplitude (∆ *F*_340_/*F*_380_), shown in **b**, and the time constant *τ* (s), which is shown in **c**. **d** Representative scatter plots from flow cytometry analysis detecting apoptosis in transfected SU-DHL-4 cells stained with Annexin V-FITC and 7-AAD. Cells were treated for 2 h with 10 µM BIRD-2, after which apoptotic cell death was detected by measuring the Annexin V-FITC-positive fraction. **e** Quantitative analysis of four independent experiments detecting apoptosis in mock-transfected SU-DHL-4 cells or cells transfected with scrambled siRNA or with STIM1 siRNA. Cells were treated for 2 h with 10 µM BIRD-2. In the scatter plots, data are represented as the mean ± SEM of at least three independent experiments. Statistically significant differences were determined with a one-way ANOVA. NS not significant.

Next, we targeted SOCE using small interfering RNA (siRNA) against STIM1 (Fig. 4a). Mock-transfected SU-DHL-4 cells and cells transfected with scrambled siRNA were used as control conditions. Compared to mock-transfected cells, the expression level of STIM1 was for 60% reduced in cells transfected with STIM1 siRNA, but not affected by scrambled siRNA (Fig. 4b). Next, it was validated that STIM1 knockdown inhibited Ca^2+^ influx after store depletion (Fig. 4c), as done before. Compared to the control conditions, the peak amplitude, used to quantify SOCE, was significantly reduced in cells transfected with siRNA against STIM1 (Fig. 4d). On the other hand, the AUC, used to quantify the TG-releasable Ca^2+^, was not reduced by knockdown of STIM1 (Fig. 4e), indicating that STIM1 siRNA selectively blocks SOCE without affecting the ER Ca^2+^-store content. Next, it was determined whether STIM1 knockdown affects the BIRD-2-induced Ca^2+^ and cell death response. The cytosolic Ca^2+^ rise provoked by 10 µM BIRD-2 was comparable between SU-DHL-4 cells transfected with STIM1 siRNA, scrambled siRNA and mock-transfected cells (Fig. 5a), indicated by very similar peak amplitudes (Fig. 5b) and time constants (Fig. 5c). Moreover, apoptotic cell death triggered by 2 h treatment with 10 µM BIRD-2 was comparable for all conditions (Fig. 5d). About 40% of the SU-DHL-4 cell population was already dead after transfection by electroporation (Fig. 5e). Upon treatment with 10 µM BIRD-2, apoptotic cell death was increased to approximately 60% in mock-transfected cells as well as in cells transfected with scrambled and STIM1 siRNA. These data further underpin that SOCE is not involved in BIRD-2-induced apoptosis in SU-DHL-4 cells.

### BIRD-2-induced cell death depends on Ca^2+^ present in the extracellular environment

**Fig. 6:**
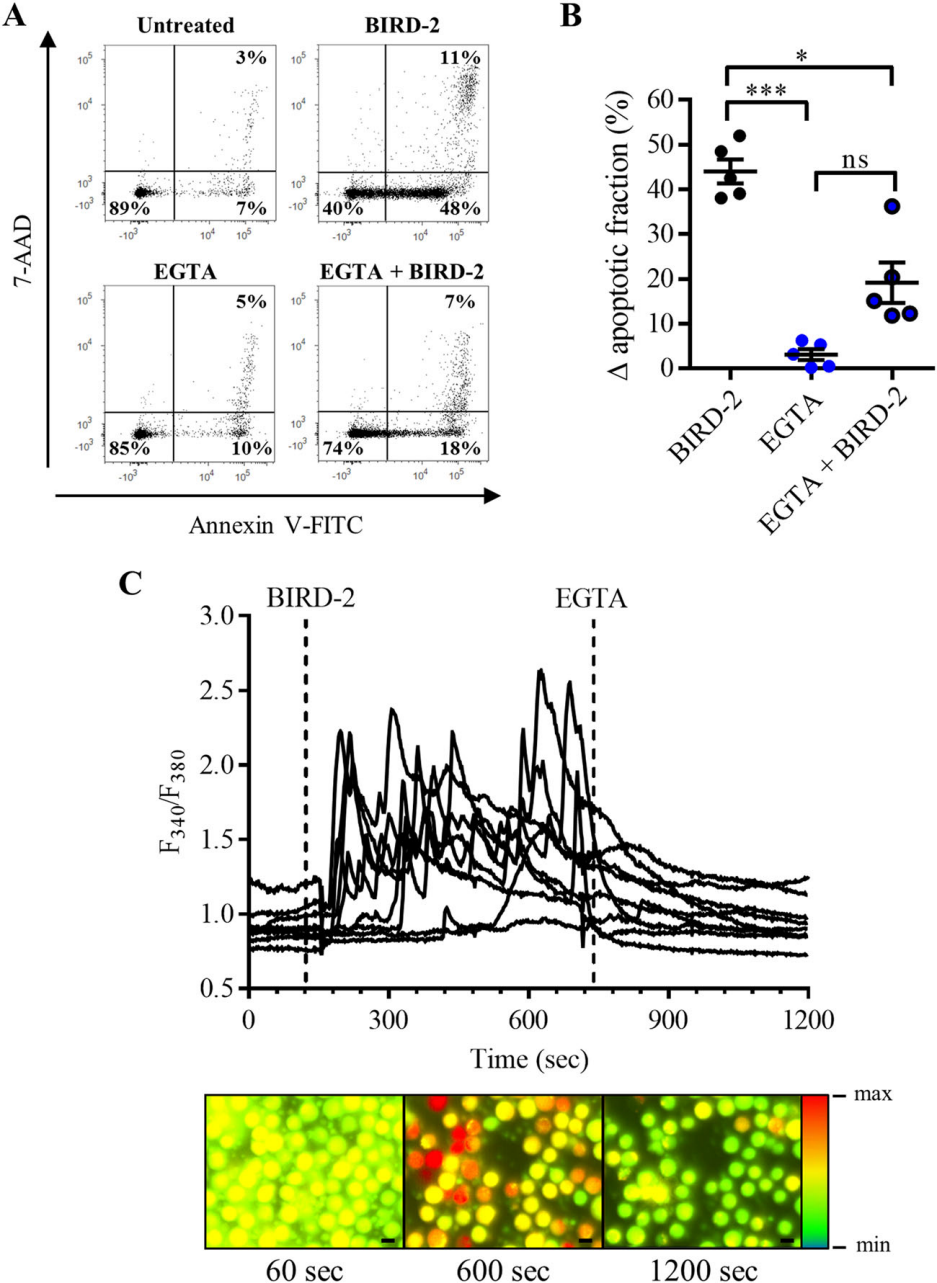
BIRD-2-triggered cell death is reduced by chelating extracellular Ca^2+^ with EGTA in SU-DHL-4 cells. **a** Representative scatter plots from flow cytometry analysis detecting apoptosis in SU-DHL-4 cells stained with Annexin V-FITC and 7-AAD. Cells were pre-treated with or without 3 mM EGTA 30 min prior to application of 10 µM BIRD-2. After 2 h of BIRD-2 treatment, apoptotic cell death was detected by measuring the Annexin V-FITC-positive fraction. **b** Quantitative analysis detecting apoptosis in SU-DHL-4 cells treated for 2 h with 10 µM BIRD-2, with or without a pre-treatment of 30 min with 3 mM EGTA. The ∆ apoptotic fraction is plotted, which corresponds to the apoptotic fraction corrected for the percentage of apoptosis detected in untreated cells. Data are represented as the mean ± SEM of five independent experiments. Statistically significant differences were determined with a one-way ANOVA (*p < 0.05, ***p < 0.001). **c** Single-cell cytosolic Ca^2+^ signals detected in SU-DHL-4 cells using the ratiometric Ca^2+^ indicator Fura-2 AM. The addition of 10 µM BIRD-2 and 3 mM EGTA are indicated by the dotted lines. Representative pseudo-color images before and after BIRD-2/EGTA addition are shown. The pseudo-color gradient at the right of the panel indicates an increasing fluorescence ratio. NS not significant.

Next, it was examined whether BIRD-2-induced apoptosis solely depends on intracellular Ca^2+^ by chelating Ca^2+^ from the extracellular environment with ethylene glycol tetraacetic acid (EGTA). Therefore, SU-DHL-4 cells were treated with 3 mM EGTA 30 min prior to the addition of 10 µM BIRD-2. After 2 h of BIRD-2 treatment, apoptotic cell death was measured via Annexin V-FITC and 7-AAD staining of the cells (Fig. 6a). BIRD-2 killed approximately 45% of the cell population, whereas treatment with only EGTA was not toxic for the cells (Fig. 6b). Interestingly, BIRD-2-triggered apoptosis was significantly reduced to approximately 20% in SU-DHL-4 cells pre-treated with EGTA, indicating BIRD-2-induced cell death depends on extracellular Ca^2+^. Next, we determined whether chelating extracellular Ca^2+^ also affects the cytosolic Ca^2+^ response provoked by BIRD-2. Therefore, single-cell Ca^2+^ levels were monitored in Fura-2 AM-loaded cells by fluorescence microscopy. Addition of 10 µM BIRD-2 provoked cytosolic Ca^2+^ oscillations in the SU-DHL-4 cells (Fig. 6c). However, treating the cells with 3 mM EGTA immediately suppressed these oscillations and caused a rapid decline in the cytosolic Ca^2+^ levels. These results indicate that, although SOCE is not involved, the cell death-inducing characteristics of BIRD-2 do depend on intracellular Ca^2+^ oscillations that are driven by Ca^2+^ influx from the extracellular environment, confirming previous results obtained in human myeloma cell lines^14^.

### BIRD-2-triggered apoptosis depends on the ER Ca^2+^-store content

**Fig. 7:**
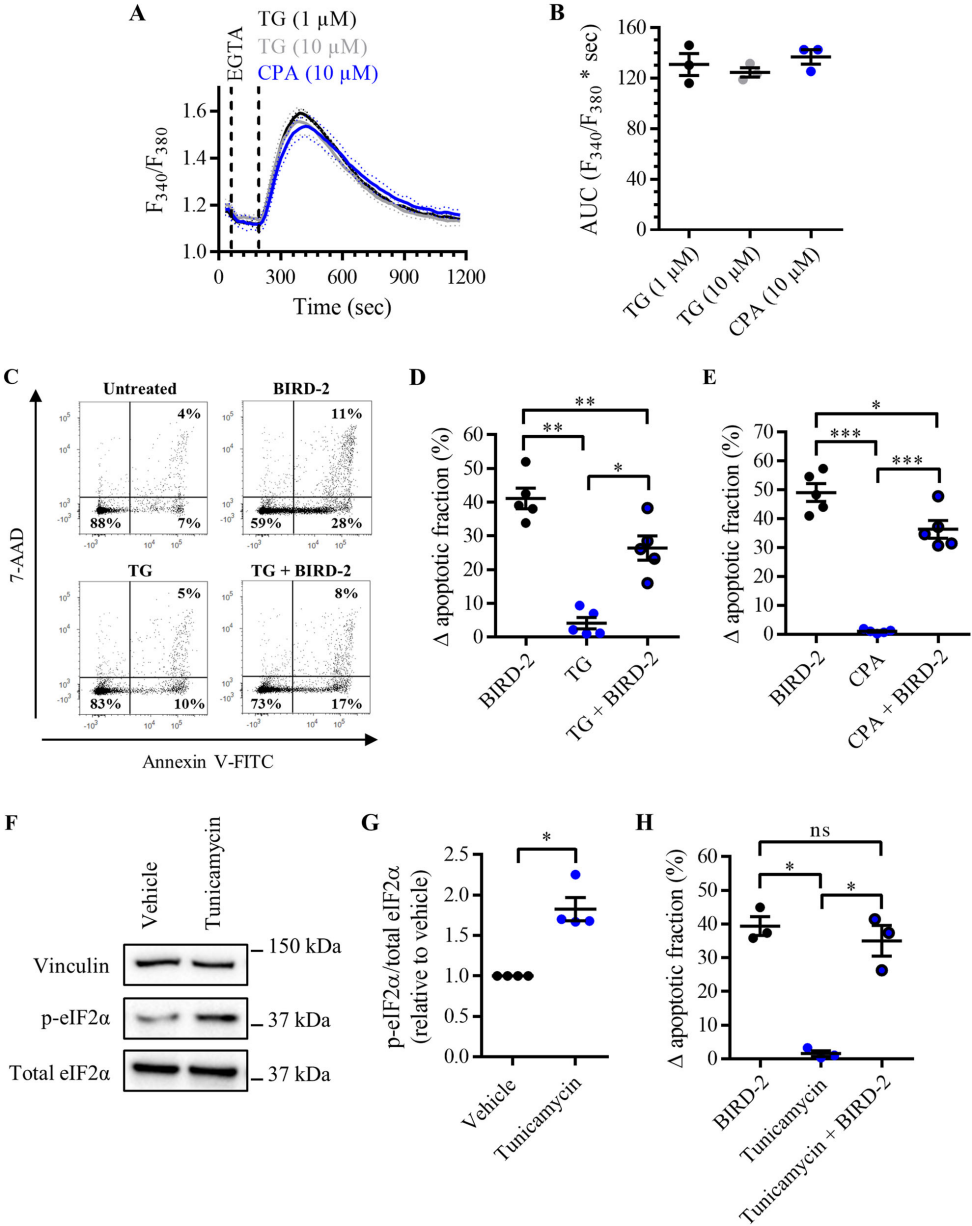
BIRD-2-triggered apoptosis is reduced by depleting the ER Ca^2+^ store in SU-DHL-4 cells. **a** Analysis of the cytosolic Ca^2+^ response in SU-DHL-4 cells using Fura-2 AM. After the addition of 3 mM EGTA, 1 or 10 µM TG or 10 µM CPA were added to deplete the ER Ca^2+^ store. The curves represent the mean ± SEM of three independent experiments. The TG/CPA-releasable Ca^2+^ is quantified by measuring the AUC (*F*_340_/*F*_380_ × s), which is shown in **b**. **c** Representative scatter plots from flow cytometry analysis detecting apoptosis in SU-DHL-4 cells stained with Annexin V-FITC and 7-AAD. Cells were pre-treated with or without 1 µM TG 30 min prior to application of 10 µM BIRD-2. After 2 h of BIRD-2 treatment, apoptotic cell death was detected by measuring the Annexin V-FITC-positive fraction. **d** Quantitative analysis of five independent experiments detecting apoptosis in SU-DHL-4 cells treated for 2 h with 10 µM BIRD-2, with or without a pre-treatment of 30 min with 1 µM TG. **e** Quantitative analysis of five independent experiments detecting apoptosis in SU-DHL-4 cells treated for 2 h with 10 µM BIRD-2, with or without a pre-treatment of 30 min with 10 µM CPA. **f** A representative western blot of four independent experiments showing the expression levels of total eIF2α and p-eIF2α in SU-DHL-4 cells treated for 4 h with 5µg/ml tunicamycin. The expression level of vinculin was used as a loading control. **g** Quantification of the p-eIF2α/total eIF2α-protein levels in SU-DHL-4 relative to vehicle-treated cells, which was set at 1. **h** Quantitative analysis of three independent experiments detecting apoptosis in SU-DHL-4 cells treated for 2 h with 10 µM BIRD-2, with or without a pre-treatment of 4 h with 5µg/ml tunicamycin. In **d**, **e**, and **h** the ∆ apoptotic fraction is plotted, which corresponds to the apoptotic fraction corrected for the percentage of apoptosis detected in untreated cells. In the dot plots, data are represented as the mean ± SEM of at least three independent experiments. Statistically significant differences were determined with a one-way ANOVA (*p < 0.05, **p < 0.01, ***p < 0.001). NS not significant

Finally, we determined whether the protection against BIRD-2-induced cell death by DPB162-AE was due to the effects of this compound on the ER Ca^2+^ content. If so, SERCA inhibitors TG and cyclopiazonic acid (CPA), which cause ER Ca^2+^-store depletion, should mimic the effects of DPB162-AE and thus suppress BIRD-2-induced cell death. To determine the ability of these compounds to trigger ER Ca^2+^ depletion, the cytosolic Ca^2+^ responses were measured in Fura-2 AM-loaded SU-DHL-4 cells upon addition of 1 and 10 µM TG and 10 µM CPA (Fig. 7a). The AUC was comparable in the three conditions, indicating that 1 µM TG and 10 µM CPA are sufficient to completely deplete the ER (Fig. 7b). Subsequently, the effect of ER depletion by TG on BIRD-2-induced apoptosis in SU-DHL-4 cells was determined (Fig. 7c). Therefore, cells were pre-treated for 30 min with 1 µM TG, after which 10 µM BIRD-2 was added. BIRD-2 (10 µM, 2 h) induced approximately 40% of apoptosis, whereas TG by itself only induced toxicity in 5% of the cell population (Fig. 7d). Interestingly, BIRD-2-triggered cell death was significantly reduced to approximately 25% in SU-DHL-4 cells pre-treated with TG. Comparable results were obtained in cells pre-treated with 10 µM CPA instead of TG (Fig. 7e), indicating that the ER Ca^2+^ content is a critical determinant for BIRD-2-provoked cell death. To exclude that TG and CPA conferred protection against BIRD-2 through ER stress and the subsequent unfolded protein response (UPR), which occur upon SERCA inhibition/ER Ca^2+^-store depletion, another ER-stress inducer that did not act through ER Ca^2+^ dysregulation was used. Therefore, we applied tunicamycin, which induces ER stress and activates the UPR by blocking N-linked glycosylation. Treatment for 4 h with 5 µg/ml tunicamycin triggered ER stress, which was observed as an increase in p-eIF2α, whereas total eIF2α levels remained unaffected (Fig. 7f, g). Next, it was determined whether tunicamycin affects BIRD-2-induced apoptosis in SU-DHL-4 cells. Pre-treatment for 4 h with 5 µg/ml tunicamycin did not protect against BIRD-2-provoked cell death (Fig. 7h), suggesting that ER stress/UPR activation was not responsible for the TG-mediated cell death protection against BIRD-2. In summary, these data indicate that the ER Ca^2+^ content is a critical determinant of BIRD-2-induced cell death in SU-DHL-4 DLBCL cells.

## Discussion

The main finding of this study is that cell death induced by BIRD-2, a peptide tool antagonizing the function of the anti-apoptotic protein Bcl-2 at the ER, is dependent on both the ER Ca^2+^-store content and extracellular Ca^2+^, but not on Ca^2+^ influx through SOCE. BIRD-2 alleviates Bcl-2's inhibitory action on the IP_3_R by targeting the BH4 domain of Bcl-2. In this way, BIRD-2 triggers Ca^2+^-mediated apoptotic cell death in cancer cells that depend for their survival on Bcl-2's anti-apoptotic function at the ER, such as the SU-DHL-4 DLBCL cells used in this study. Our results indicate that Ca^2+^ present in the extracellular environment as well as the ER Ca^2+^ levels both critically contribute to BIRD-2-induced cell death in DLBCL cancer cells.

First, we investigated whether SOCE, an important Ca^2+^ influx pathway activated upon ER-store depletion, is involved in BIRD-2-induced cell death, since BIRD-2 triggers apoptotic cell death through excessive Ca^2+^ release from the ER. Furthermore, the key SOCE players STIM and Orai have been implicated in the control of cell death and survival pathways^27,28^. The contribution of Ca^2+^ influx through STIM/Orai signaling to BIRD-2-induced cell death was determined by using pharmacological SOCE inhibitors. One of the compounds used was DPB162-AE, a 2-APB analog developed as a more potent and more selective SOCE inhibitor compared to 2-APB^29^. Previous work from our lab showed that DPB162-AE is indeed a very potent inhibitor of SOCE, although it also affected the ER Ca^2+^ content^25^. At concentrations required for adequate SOCE inhibition, DPB162-AE depleted the ER Ca^2+^ store by inducing an ER Ca^2+^-leak pathway. Here, we showed that DPB162-AE treatment reduced the BIRD-2-induced pro-apoptotic Ca^2+^ signaling and cell death responses in SU-DHL-4 DLBCL cells. However, it is not clear whether this protection was the result of SOCE inhibition, since DPB162-AE also depletes the ER Ca^2+^ store. Because of this, YM-58483 and GSK-7975A, two SOCE inhibitors that do not alter the ER Ca^2+^ content, were used to examine the contribution of SOCE to BIRD-2-triggered apoptosis. Both compounds potently inhibited SOCE in SU-DHL-4 cells, without reducing ER Ca^2+^ levels. Surprisingly, SOCE inhibition with these pharmacological tools did neither reduce BIRD-2-induced cell death nor suppress the BIRD-2-triggered cytosolic Ca^2+^ response. Furthermore, these results were confirmed by knocking down STIM1 with siRNA, which also blocked SOCE without affecting ER Ca^2+^ levels. Hence, these data indicate that SOCE does not contribute to BIRD-2-induced cell death.

There are several plausible reasons why SOCE may not be activated upon BIRD-2 application, despite the fact that BIRD-2 provokes Ca^2+^ oscillations that originate from the ER. First, it might be that BIRD-2 treatment does not completely deplete the ER Ca^2+^ store, since BIRD-2 does not inhibit SERCA activity. As a consequence, the SERCA pump might partially refill the ER Ca^2+^ store after treatment with BIRD-2, thus evading complete depletion of the ER Ca^2+^ store. Second, besides SOCE through STIM/Orai signaling, other Ca^2+^-influx pathways might be stimulated upon BIRD-2 application. For example, transient receptor potential (TRP) channels, more particularly TRPC channels, have been proposed to be activated by ER store depletion^30,31^. Third, ectopic Bcl-2 overexpression has been implicated in downregulation of SOCE in both HeLa and LNCaP prostate cancer epithelial cells, which could be attributed to Bcl-2's ability to reduce ER Ca^2+^ loading^32,33^. Recent work confirmed that overexpression of wild-type Bcl-2 resulted in decreased SOCE, though accompanied by an increase in steady-state ER Ca^2+^ levels, protecting against TG-induced cell death^34^. This Bcl-2 property could be abrogated by the α5-helical Bcl-2 mutant 144WGR146/144AAA146, which partially depleted ER Ca^2+^ stores by itself and boosted SOCE after TG treatment, resulting in aggravated TG-induced cell death. Yet, it remains unclear whether such SOCE modulation can occur by endogenously elevated Bcl-2 proteins, such as in the B-cell cancer cell model used here. Importantly, in this study we showed that Ca^2+^ present in the extracellular environment contributes to BIRD-2 toxicity, as chelating extracellular Ca^2+^ with EGTA conferred protection against BIRD-2-provoked cell death in SU-DHL-4 cells. Moreover, the cytosolic Ca^2+^ oscillations provoked by BIRD-2 treatment were abruptly suppressed by the addition of the extracellular Ca^2+^ chelator. Hence, although SOCE is not activated by the peptide tool, our results indicate that BIRD-2 does not solely depend on the intracellular Ca^2+^ levels for its pro-apoptotic effects. This study revealed a hitherto unappreciated role for extracellular Ca^2+^ in BIRD-2-induced cell death. Yet, the exact molecular mechanisms by which extracellular Ca^2+^ enters BIRD-2-sensitive cells and contributes to BIRD-2-induced cell death remain elusive.

Besides extracellular Ca^2+^, the ER Ca^2+^ stores also determine apoptotic cell death mediated by BIRD-2 in DLBCL cancer cells, as we previously showed that IP_3_R inhibition suppresses BIRD-2 sensitivity^12^. Indeed, treatment of SU-DHL-4 cells with the SERCA inhibitors TG and CPA protected against BIRD-2-induced cell death. As such, we hypothesize that the protective effects of DPB162-AE against BIRD-2-induced cell death are due to the ability of DPB162-AE to lower ER Ca^2+^ levels and not due to its SOCE-inhibitory properties. Importantly, the observed protection is a direct consequence of ER Ca^2+^-store depletion, and is not caused by ER stress/UPR activation, since tunicamycin, an ER-stress inducer that does not directly target the Ca^2+^-signaling machinery, could not reduce BIRD-2-induced apoptosis. Hence, our work implicates that the proper filling of the ER Ca^2+^ stores is a critical factor underlying the susceptibility of DLBCL cancer cells towards BIRD-2 exposure. This is in line with previous work showing that the ER Ca^2+^-filling state is a critical determinant in the apoptotic sensitivity of cells towards cell death inducers^35,36^.

We had previously shown that BIRD-2-induced apoptotic cell death in DLBCL cancer cells depends on multiple cellular determinants. First, DLBCL cells with high expression levels of IP_3_R2, the isoform with the highest sensitivity to its ligand IP_3_, are very sensitive to BIRD-2, whereas cells with low IP_3_R2 expression levels are less susceptible to BIRD-2-induced cell death^12^. Second, we have recently shown that constitutive IP_3_ signaling is an additional determinant of the sensitivity of B-cell cancers, including DLBCL and CLL, to BIRD-2^17^. Constitutive IP_3_ signaling has a pro-survival role in cancer cells, but it can be switched into pro-death signaling by disturbing Bcl-2/IP_3_R-complex formation with BIRD-2. Finally, here we show that Ca^2+^-mediated cell death induced by BIRD-2 depends on the Ca^2+^ levels of the ER as well as on the presence of Ca^2+^ in the extracellular environment, but not on Ca^2+^ influx via STIM/Orai signaling. Thus, our data indicate that both extracellular Ca^2+^ and the ER Ca^2+^-store content contribute to cell death induced by BIRD-2.

## Materials and methods

### Cell culture

DLBCL SU-DHL-4 cells were kindly provided by Dr. A. Letai (Dana-Farber Cancer Institute, USA) and cultured in suspension in RPMI-1640 medium (Invitrogen, Merelbeke, Belgium). Human cervical carcinoma HeLa cells were cultured in Dulbecco's modified Eagle's medium medium (Invitrogen, Merelbeke, Belgium). All media were supplemented with 10% heat-inactivated fetal bovine serum, l-glutamine (100× GlutaMAX, Gibco/Invitrogen, Merelbeke, Belgium) and penicillin and streptomycin (100× Pen/Strep, Gibco/Invitrogen, Merelbeke, Belgium). Cells were cultured at 37 °C and 5% CO_2_. All human cell lines used in this study have been authenticated using autosomal short tandem repeat profiling performed by the University of Arizona Genetics Core and fully matched the DNA fingerprint present in reference databases.

### Antibodies and reagents

The following antibodies were used for immunoblotting: anti-GAPDH antibody (Sigma-Aldrich, Munich, Germany, G8795), anti-STIM1 antibody (Abnova, Taipei, Taiwan, PAB12017), anti-total eukaryotic initiation factor 2α (eIF2α) antibody (Thermo Scientific, Waltham, MA, USA, AHO0802), anti-phospho-eIF2α (p-eIF2α) antibody (Thermo Scientific, Waltham, MA, USA, MA5-15133), anti-vinculin antibody (Sigma-Aldrich, Munich, Germany, V9131). Reagents were as follows: EGTA (Acros Organics, Geel, Belgium, 409910250), TG (Alomone labs, Jerusalem, Israel, T-650), CPA (Enzo Life Sciences, Farmingdale, NY, USA, BML-CA415-0010), calcium chloride (CaCl_2_) (Merck, Darmstadt, Germany, 102382), Fura-2 AM (Life Technologies, Carlsbad, CA, USA, F1221), tunicamycin (Sigma-Aldrich, Munich, Germany, T7765), and YM-58483 (Abcam, Cambridge, UK, ab144413). GSK-7975A was a gift from GlaxoSmithKline (Stevenage, UK). DPB162-AE was kindly provided by Dr. K. Mikoshiba (Brain Science Institute, RIKEN, Tokyo, Japan). BIRD-2 peptide (sequence: RKKRRQRRRGGNVYTEIKCNSLLPLAAIVRV) was purchased from LifeTein (South Plainfield, NJ, USA) with a purity of >85%.

### siRNA transfection

Sequences for siRNA were: STIM1 siRNA, 5′-GGAGGAUAAUGGCUCUAUUdTdT-3′ and scrambled siRNA, 5′-AAUUCUCCGAACGUGUCACdTdT-3′. SU-DHL-4 cells were transfected by electroporation using AMAXA Kit V (Lonza, Basel, Switzerland, VCA-1003) program P-005. Briefly, 5 × 10^6^ cells were transfected with 2 µg STIM1 siRNA or scrambled siRNA. After transfection, cells were seeded at 1 × 10^6^ cells/ml and approximately 36 h post-transfection the change in gene expression was measured by western blot analysis.

### Cytosolic Ca^2+^ measurements in intact cell populations

Cytosolic Ca^2+^ levels were monitored with Fura-2 AM as previously described^12,37^. Briefly, Fura-2 AM measurements were performed by seeding the SU-DHL-4 cells in 96-well plates (Greiner) with poly-l-lysine coating. The cells were loaded for 30 min with 1.25 µM Fura-2 AM at room temperature in modified Krebs solution (containing 150 mM NaCl, 5.9 mM KCl, 1.2 mM MgCl_2_, 11.6 mM HEPES (pH 7.3), 11.5 mM glucose and 1.5 mM CaCl_2_), followed by a de-esterification step of 30 min in the absence of Fura-2 AM. During the de-esterification cells were treated with vehicle, 3 µM YM-58483, 3 µM GSK-7975A, or 10 µM DPB162-AE. Fluorescence was monitored on a FlexStation 3 microplate reader (Molecular Devices, Sunnyvale, CA, USA) by alternately exciting the Ca^2+^ indicator at 340 and 380 nm and collecting emitted fluorescence at 510 nm. EGTA (final concentration 3 mM), TG (final concentration 1 µM), CaCl_2_ (final concentration 10 mM), or BIRD-2 (final concentration 10 µM) were added as indicated. All traces are shown as the ratio of emitted fluorescence of Fura-2 (*F*_340_/*F*_380_).

### Single-cell cytosolic Ca^2+^ imaging

Single-cell cytosolic Ca^2+^ measurements were performed as previously described^38^. In brief, SU-DHL-4 cells were loaded with Fura-2 AM as described above. Single-cell imaging was performed using a Zeiss Axio Observer Z1 Inverted Microscope equipped with a 20× air objective and a high-speed digital camera (Axiocam Hsm, Zeiss, Jena, Germany). Data are shown as the ratio of emitted fluorescence of Fura-2 (*F*_340_/*F*_380_).

### Single-cell ER Ca^2+^ imaging

Single-cell ER Ca^2+^ measurements were performed as previously described^39^. ER Ca^2+^ levels were measured with the genetically encoded Ca^2+^ indicator G-CEPIA1*er*, which was kindly provided by Dr. M. Iino (The University of Tokyo, Tokyo, Japan)^26^. The G-CEPIA1*er* construct was introduced into HeLa cells utilizing X-tremeGENE HP DNA transfection reagent (Roche, Mannheim, Germany, 06366546001) according to the manufacturer’s protocol. A Zeiss Axio Observer Z1 Inverted Microscope equipped with a 20× air objective and a high-speed digital camera (Axiocam Hsm, Zeiss, Jena, Germany) were used for these measurements. Changes in fluorescence were monitored in the GFP channel (480/520 nm excitation/emission). To chelate extracellular Ca^2+^, EGTA was added to a final concentration of 3 mM. One minute later, 1 µM TG or different SOCE inhibitors were added. All traces were normalized (*F*/*F*_0_) where *F*_0_ is the starting fluorescence of each trace.

### ATPase activity measurements

Ca^2+^-dependent ATPase activity experiments were performed on microsomes of COS cells overexpressing SERCA2b using an endpoint colorimetric assay responding to the released inorganic phosphate, as previously described^40^. The SOCE inhibitors were pre-incubated with the microsomes for 30 min at 4 °C, followed by 5 min at 37 °C. The Ca^2+^-dependent ATPase activity was measured at maximal (free [Ca^2+^] 3.16 µM) and submaximal (free [Ca^2+^] 0.316 µM) [Ca^2+^].

### Apoptosis assay

SU-DHL-4 cells (5 × 10^5^ cells/ml) were treated for 2 h with 10 µM BIRD-2 with or without a pre-treatment of 30 min with 3 µM YM-58483, 3 µM GSK-7975A, 10 µM DPB162-AE, 3 mM EGTA, 1 µM TG, 10 µM CPA, or 5 µg/ml tunicamycin. Subsequently, cells were pelleted by centrifugation and incubated with Annexin V-FITC (Life Technologies, Carlsbad, CA, USA, V13245) and 7-AAD (Becton Dickinson, Franklin Lakes, NJ, USA, 555815). Cell suspensions were analyzed with an Attune Acoustic Focusing Flow Cytometer (Applied Biosystems). Cell death by apoptosis was scored by quantifying the population of Annexin V-FITC-positive cells using the FlowJo version 10 software. Data are plotted as the ∆ apoptotic fraction (=apoptotic fraction in treated cells − apoptotic fraction in untreated cells).

### Western blot analysis

SU-DHL-4 cells were washed with phosphate-buffered saline and incubated at 4 °C with lysis buffer (20 mM Tris-HCl (pH 7.5), 150 mM NaCl, 1.5 mM MgCl_2_, 0.5 mM dithiothreitol, 1% Triton-X-100, a phosphatase inhibitor cocktail tablet (PhosSTOP, Roche, Mannheim, Germany) and 1 tablet complete EDTA-free protease inhibitor (Thermo Scientific, Brussels, Belgium)) for 30 min on a head-over-head rotor. Cell lysates were centrifuged for 5 min at 4000 × g and analyzed by western blotting as previously described^41^.

### Statistical analysis

Results are expressed as the mean ± SEM. The number of independent experiments is always indicated. Significance was determined using a two-tailed paired Student’s *t* test or a one-way analysis of variance (ANOVA) with a post hoc Tukey’s test, as appropriate. Differences were considered significant at *p* < 0.05.
